# Ferroptosis and Its Modulation by Autophagy in Light of the Pathogenesis of Lysosomal Storage Diseases

**DOI:** 10.3390/cells10020365

**Published:** 2021-02-10

**Authors:** Karolina Pierzynowska, Estera Rintz, Lidia Gaffke, Grzegorz Węgrzyn

**Affiliations:** Department of Molecular Biology, University of Gdansk, Wita Stwosza 59, 80-308 Gdansk, Poland; estera.rintz@ug.edu.pl (E.R.); lidia.gaffke@ug.edu.pl (L.G.); grzegorz.wegrzyn@biol.ug.edu.pl (G.W.)

**Keywords:** ferroptosis, programmed cell death, autophagy-dependent ferroptosis, lysosomal storage diseases

## Abstract

Ferroptosis is one of the recently described types of cell death which is dependent on many factors, including the accumulation of iron and lipid peroxidation. Its induction requires various signaling pathways. Recent discovery of ferroptosis induction pathways stimulated by autophagy, so called autophagy-dependent ferroptosis, put our attention on the role of ferroptosis in lysosomal storage diseases (LSD). Lysosome dysfunction, observed in these diseases, may influence ferroptosis efficiency, with as yet unknown consequences for the function of cells, tissues, and organisms, due to the effects of ferroptosis on physiological and pathological metabolic processes. Modulation of levels of ferrous ions and enhanced oxidative stress, which are primary markers of ferroptosis, are often described as processes associated with the pathology of LSD. Inhibition of autophagy flux and resultant accumulation of autophagosomes in neuronopathic LSD may induce autophagy-dependent ferroptosis, indicating a considerable contribution of this process in neurodegeneration. In this review article, we describe molecular mechanisms of ferroptosis in light of LSD, underlining the modulation of levels of ferroptosis markers in these diseases. Furthermore, we propose a hypothesis about the possible involvement of autophagy-dependent ferroptosis in these disorders.

## 1. Introduction

Ferroptosis is one of the recently described types of cell death which differs from other well-known types of regulated cell death (apoptosis, necrosis, necroptosis, and others) morphologically, physiologically, and biochemically. Many of its characteristic features, as well as its activation pathways, are still being discovered, but generally, this process is characterized by the accumulation of reactive oxygen species (ROS) as products of increased efficiency of iron metabolism and lipid peroxidation [1,2,3]. 

The ferroptosis process, as one of regulated cell death, has been known since 2012 [1,4]. It consists of various changes in cellular functions associated with cell morphology, physiology, and biochemistry. In Table 1, crucial ferroptosis features are summarized and compared to the most frequent programmed cell death—apoptosis.

The exact role of ferroptosis has just begun to be the center of interest of researchers. Studies performed to date have indicated its significant role in a few human diseases, including its increased efficiency in neurodegenerative diseases, ischemic reperfusion injury, atherosclerosis, and cancer [2]. However, the efficiency of this process, pathways of its activation, and its role in the development of most diseases remain largely unknown in many disorders. Recently discovered pathways link the autophagy process (lysosomal degradation of macromolecules) and ferroptosis.

In the autophagy process, a degradation-desired macromolecule is engulfed by a membrane called phagophore, forming an autophagosome. This vesicle is fused with lysosome, and lysosomal acid hydrolases digest its content. Autophagy is a physiologically important process which recycles misfolded molecules or dysfunctional organelles, forming precursors of newly synthesized macromolecules. However, too intensive autophagy may also lead to cell death. In fact, increased efficiency of autophagy was observed in response to ferroptosis inductors [3]. These discoveries inspired us to put attention on lysosomal storage diseases (LSD) as disorders in which modulation of ferroptosis might be involved in their molecular pathomechanism.

LSD are a group of 50 or so inherited metabolic diseases caused by mutations in genes encoding lysosomes functions [4]. Dysfunctions in lysosomal ability to convert biologically significant polymers into oligomers and monomers result in the storage of undegraded or partially degraded compounds inside these organelles [4,5]. When considering the molecular mechanisms of LSDs, one can distinguish four major defects leading to these disorders: (i) inactivation of one of the specific lysosomal hydrolases (sphingolipids, glycoproteins, glycosaminoglycans); (ii) defect in a protein involved in the transport of particular compounds through lysosomal membranes; (iii) inactivation of the enzyme that modifies lysosomal proteins, ensuring their proper localization and function; and (iv) lack of specific activators for lysosomal enzymes [6]. Another classification of LSD is based on the nature of stored compound(s). Therefore, the following subgroups of LSDs can be listed: (i) mucopolysaccharidoses, characterized by the accumulation of glycosaminoglycans; (ii) oligosaccharidoses, characterized by the accumulation of oligosaccharides (e.g., mannosidoses, fucosidosis); (iii) glycogen storage diseases (e.g., Pompe disease, Danon disease); (iv) lipidoses in which sphingomyelin is stored (e.g., Niemann–Pick disease); (v) neuronal ceroid lipofuscinoses, characterized by the accumulation of lipopigments; (vi) mucolipidoses, characterized by the accumulation of combinations of lipids and carbohydrates; (vii) sphingolipidoses, characterized by defects of the degradation of lipids containing ceramide (e.g., gangliosidoses GM1 and GM2, Fabry disease, Krabbe disease, metachromatic leukodystrophy, Gaucher disease); and (viii) other diseases in which specific compounds accumulate, such as cystinosis (accumulation of cystine) or Salla disease (accumulation of sialic acid) [7].

Although LSDs are monogenic diseases, specific therapeutic options are available only for relatively few of them. Bone marrow or hematopoietic stem cell transplantations were used in various LSDs; however, their efficacy was very different in particular diseases, and even if some efficacy was observed, it occurred only when the procedure was performed early in life, before the age of 2 years. Substrate reduction therapy, a procedure based on the inhibition of synthesis of a compound which cannot be degraded in lysosomes, is currently available for Gaucher disease and Niemann–Pick disease type C, while studies on the use of small molecules interfering with the syntheses of various compounds are ongoing. Enzyme replacement therapy (intravenous administration of recombinant human enzyme which is otherwise deficient in a patient’s cells) is the most often used specific treatment of LSD, and is currently available for several diseases, including Gaucher disease, Fabry disease, Pompe disease, and mucopolysaccharidoses types I, II, IVA, VI, and VII. However, this therapy is inefficient in treatment of the central nervous system, since intravenously administered enzyme cannot efficiently cross the blood–brain barrier. Finally, gene therapy is a promising option, and there are many studies ongoing which are focused on the use of this procedure in LSD; however, to date, no gene therapy for this group of diseases has been registered [8,9,10].

## 2. Molecular Mechanisms of Ferroptosis

Determining the exact molecular content of the induction of ferroptosis is one of the challenges posed to cell biologists. Studies of recent years indicated that this process is primarily dependent on (i) iron levels in the cell, (ii) specific metabolic pathways, (iii) the GPX4-dependent pathway, and (iv) ROS levels and lipid peroxidation. Moreover, it can also be regulated by (v) MAP kinases and/or (vi) the intensity of autophagy (in the process called autophagy-dependent ferroptosis) [1,3].

Ferroptosis is primarily dependent on iron concentration [4]. Circulating iron occurs as ferritin-bound Fe^3+^ ions. Following import to cells by the transferrin receptor TFR1, it is delivered to the endosome where ferrireductase STEAP3 catalyzes the reduction of Fe^3+^ to Fe^2+^. The SLC11A2 transporter liberates Fe^2+^ from the endosome to the cytoplasm where iron excess is stored in the ferritin-bound form, while in the lack of such storage, the Fenton reaction leads to the formation of ROS which are involved in lipid peroxidation, thus inducing ferroptosis. Ferroportin SLC11A3 converts Fe^2+^ to Fe^3+^ and participates in iron export from cells [4].

One more pathway that can induce ferroptosis by modulating iron levels is the frataxin-dependent pathway. Lowering the level of this protein results in mitochondrial dysfunction, which in turn leads to iron accumulation in mitochondria and enhanced efficiency of the Fenton reaction. These features may be confirmed by the results of studies on models of Friedreich’s ataxia, a disease caused by mutations in the gene encoding frataxin (the *FXN* gene), resulting in its decreased expression. An increase in the levels of ferroptosis markers in fibroblasts collected from patients with Friedreich’s ataxia (increased lipid peroxidation, decreased level of antioxidant enzymes, increased protein oxidation) has been reported [11,12]. Such studies were also performed on mouse models. Increased levels of ferroptosis markers were observed in adipocyte precursors in *FXN* knock-in/knock-out (KIKO) mice (increased lipid peroxidation and decreased glutathione peroxidase 4 activity) [13], and in C2C12 mouse myoblasts after *FXN* gene silencing (increased expression of pro-ferroptotic and decreased expression of anti-ferroptotic genes) [12,13]. Moreover, fibroblasts collected from Friedreich’s ataxia patients, as well as *FXN* knockdown human fibrosarcoma HT-1080 cells, were considerably more sensitive to the administration of erastin, one of the best characterized ferroptosis activators [11,14]. The results showing the restoration of resistance to ferroptosis of *FXN* knockdown cells after blocking the signal of iron starvation indicated the dependence of this phenomenon on intracellular iron concentration [14]. Another disease that could indicate the role of frataxin in ferroptosis is the alcoholic liver disease. Long-term administration of ethanol to mice reduced the expression of the gene encoding frataxin, leading to the accumulation of ROS and the mitochondrial iron pool in primary hepatocytes. Moreover, deficiency of frataxin enhanced ethanol-driven ferroptosis, and restoration of the appropriate level of frataxin reduced the sensitivity of liver to ethanol treatment [15].

**Figure 1:**
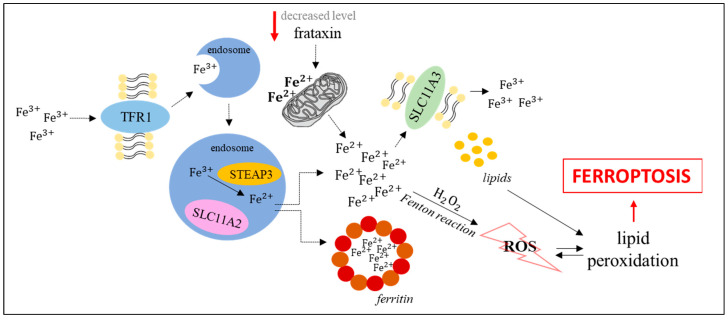
Activation of ferroptosis by the iron-dependent pathway [1,4,16,17].

The crucial role of iron in ferroptosis induction is also supported by the results of studies on overexpression of the gene coding for TLR1 and repression of the ferritin-coding gene which resulted in the stimulation of this process. Furthermore, impaired expression of the IREB2 transcription factor (that acts to regulate iron levels) caused inhibition of ferroptosis [1,16,17]. The iron-dependent pathway leading to ferroptosis, described above, is depicted in Figure 1.

**Figure 2:**
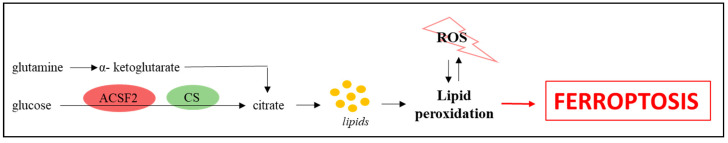
Activation of ferroptosis by metabolic processes-dependent pathways [1,4,16].

Apart from iron-dependent ROS generation (described above), enhanced lipid peroxidation can result from changes in glucose and glutamate metabolism. Acyl-CoA synthetase (ACSF2) and citrate synthase (CS), as regulators of the mitochondrial fatty-acid metabolism, participate in the biochemical pathway from glucose to citrate (through glycolysis and Krebs cycle) that can be converted to substrates for lipid synthesis, of which increased levels may lead to their peroxidation [1,16]. Moreover, conversion of glutamate to α-ketoglutarate contributes to the production of citrate [18], causing effects as described above [1,16]. In fact, knock-out mutations in genes coding for ACSF2 or CS halted ferroptosis (induced by erastin), indicating their considerable role in the stimulation of this process [1,16]. In accordance with this model, a small molecule transaminase inhibitor, aminooxyacetic acid, which blocks the conversion of glutamate to α-ketoglutarate, inhibits ferroptosis [16]. Similar effects are caused by inhibition of the pentose-phosphate pathway due to mutations in genes coding for glucose-6-phosphate dehydrogenase and phosphoglycerate dehydrogenase (Figure 2) [1,4].

Glutathione peroxidase 4 (GPX4), one of the antioxidant enzymes, is a key regulator of ferroptosis [1]. A major co-factor of GPX4 is glutathione (GSH), which can be oxidized to glutathione disulfide (GSSG), and then reduced by NADPH/H^+^-dependent GSH reductase. GSH synthesis is catalyzed by glutamate-cysteine ligase and GSH synthetase, and requires cysteine, glutamate, and glycine as substrates. The direct effect of GSH levels on ferroptosis emerged from the use of erastin (one of the ferroptotic activators), which by lowering GSH levels activates cell death identically to that caused by a lack of GPX4 [19]. So far, a few pathways have been described that could reduce GSH levels in cells, and most of them are dependent on cysteine levels. Intracellular cysteine concentration depends on the activity of cysteine/glutamate antiporter SLC7A11 (Xc^−^ system). Cysteine for GSH synthesis may also come from the degradation of macromolecules in lysosomes, as it can be released from these organelles into the cytoplasm [19]. Thus, glutamate/cysteine exchange by SLC7A11 and the high efficiency of lysosomal degradation of macromolecules are necessary to maintain an adequate level of cysteine. Its decreased level and/or reduced activities of glutathione-synthesizing enzymes can lead to decreased activity of GPX4 which, in turn, causes the accumulation of ROS and lipid peroxidation [19,20]. In fact, inhibition of GPX4 can result in increased ROS concentrations, while GPX4 overexpression cause ROS depletion, modulating ferroptosis efficiency [21]. 

A protein that has recently been reported to be involved in the regulation of ferroptosis by this pathway is Beclin-1. Under the influence of erastin and other ferroptosis activators, it can bind to the components of the Xc^−^ system, limiting cysteine/glutamate exchange, which leads to a decrease in GSH concentration, inhibition of GPX4, and, as a consequence, the induction of ferroptosis. Binding of Beclin-1 to the Xc^−^ system is determined by its phosphorylation by AMPK kinase at sites S90 and S93. Mutations of the gene coding for Beclin-1, causing modifications in the above mentioned phosphorylation sites, or a reduction in the activity of AMPK kinase, led to the inhibition of erastin-induced ferroptosis, confirming this conclusion [3,22].

NRF2 (nuclear factor erythroid 2-related factor 2) is another protein that has a large impact on the activity of the Xc^−^ system components. Since an increase in its level leads to enhanced expression of genes encoding the SLC7A11 protein and antioxidant proteins, it plays an anti-ferroptotic role. Studies carried out on tumor cell models (hepatocellular carcinoma and head and neck squamous cell carcinoma) have shown that *NRF2* overexpression reduces, while knock-out in this gene increases, cell sensitivity to ferroptosis inducers [1,23,24,25]. Reduction in the expression of the *NRF2* gene also sensitized the cells to RSL3 (one of the ferroptosis activators that is a GPX4 inhibitor) [24]. These studies also indicated that expression of the gene encoding the p62 protein is necessary to maintain high NRF2 levels after exposure to ferroptosis inducers. This determines the interaction of NRF2 with transcriptional coactivator small v-maf avian musculoaponeurotic fibrosarcoma oncogene homolog proteins, activating the transcription of genes encoding antioxidant enzymes (quinone oxidoreductase-1, heme oxygenase-1, and ferritin heavy chain-1). Silencing of the expression of the p62-coding gene, as well as genes encoding the above mentioned enzymes, led to an increase in the sensitivity of cells to erastin and sorafenib. The same effect was achieved by genetic or pharmacological silencing of the expression of *NRF2*, both in vitro and in tumor xenograft models [23]. ARF (ARF tumor suppressor, also called p14ARF) is an additional protein that is involved in the regulation of NRF2 activity, and thus has a broad impact on the effectiveness of ferroptosis. This protein, by binding to NRF2, inactivates it, which prevents the positive regulation of *SLC7A11* expression, leading to the induction of ferroptosis. A high level of expression of the *ARF* gene makes cells more sensitive to this type of cell death, while lowering of its expression promotes their survival [25]. 

The p53 protein has a large impact on the efficiency of ferroptosis, as it regulates the expression of *SLC7A11*. Recent studies have indicated that its activation by nutlin-3 reduced the expression of *SLC7A11* in HT-1080 cells, while mutations in the p53-encoding gene completely canceled this effect. Thus, activation of p53 leads to a blockage of cysteine import, and thus a reduction in GSH levels, inactivation of GPX4, and the induction of ferroptosis [26]. On the other hand, p21 (encoded by the *CDKN1A* gene) is another p53 target protein. Upregulation of p21 ensures that a high level of GSH is maintained, which is conducive to cell survival, but the exact mechanisms by which this happens are still under investigation. The recycle of oxidized GSH to reduced GSH, decreased export of GSH from the cell, or reduced consumption of GSH were reported as possible molecular mechanisms of the influence of p21 on GSH levels [27,28]. 

**Figure 3:**
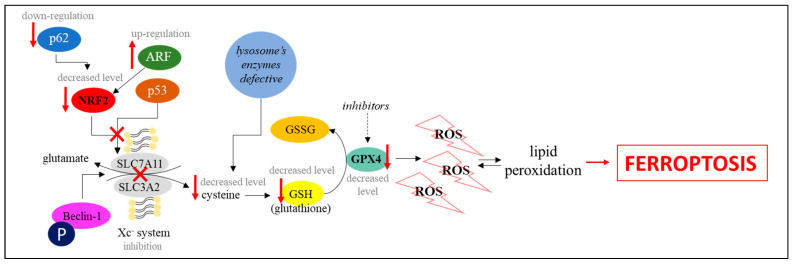
Activation of ferroptosis by GPX4-dependent pathway [1,19,20,21,22,23,24,25].

The above described GPX4-dependent pathway, leading to ferroptosis, is presented in Figure 3.

**Figure 4:**
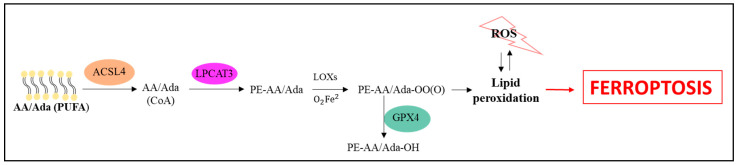
Activation of ferroptosis by ROS levels and lipid peroxidation-dependent pathway [1,3,29].

Ferroptosis induction can also result from reactions of ROS with polyunsaturated fatty acids (PUFAs) in lipid membranes. Acyl-CoA synthetase long chain family member 4 (ACSL4), lysophosphatidylcholine acyltransferase 3 (LPCAT3), and arachidonate lipoxygenase (ALOX) enzymes are involved in PUFAs metabolism. They acetylate arachidonic acid (AA) and adrenic acid (AdA), catalyze their conversion to membrane phospholipids, and then oxidize them to lipid peroxides [3,29]. Decreased levels of ACSL4 and LPCAT3 in cells prevented the oxidization of sensitive fatty acids in the membrane. On the other hand, depletion of GPX4 led to an abundance of oxidized membranes enriched with arachidonic acid. The role of arachidonic acid appears to be particularly important, as in the absence of GPX4 activity, the liberation of its metabolites (hydroxyeicosatetraenoic acids (HETE): 5-HETE, 11-HETE, and 15-HETE) was observed, in contrast to apoptosis. Thus, their appearance may be specific to ferroptosis [29]. ACSL4 is, therefore, considered as not only a protein which mediates ferroptosis induction, but also as a marker of this process [3]. This pathway, leading to ferroptosis, is presented in Figure 4.

It appears that ferroptosis may also be dependent on MAPK proteins, particularly extracellular signal-regulated kinases (ERK), p38 mitogen-activated protein kinases (p38), and c-Jun N-terminal kinases (JNK), suggesting activation of the Ras/Raf/MEK/ERK pathway. Inhibition of components of this pathway led to a reduced sensitivity to cell death caused by erastin in 12 different sarcoma cell lines [1,30,31,32]. Additional studies on the pathways involved in the induction of ferroptosis indicated the involvement of further genes, the expression of which is necessary for erastin-induced ferroptosis in two cell line models, HT-1080 and Calu-1. These genes included *RPL8* (coding for 60S ribosomal protein L8), *ATP5G3* (encoding the ATP5G3 protein), *CS* (coding for citrate synthase), *TTC35* (encoding tetratricopeptide repeat domain 35), *ACSF2* (encoding acyl-CoA synthetase family member 2), and *IREB2* (coding for iron response element binding protein 2). Silencing the expression of these genes abolished the erastin effect on ferroptosis induction in these cell lines [16,33]. *HMGB1* (coding for high mobility group box 1 protein) is another gene whose involvement in ferroptosis has been reported. The release of the HMGB1 protein after the use of ferroptosis activators, such as erastin or sorafenib, and a decrease in its secretion after the use of ferroptosis inhibitors (pharmacologically, after treatment with ferrostatin, or genetically, after silencing the expression of the *ACSL4* gene) in HT1080 and PANC1 cell lines were demonstrated [3,34]. In addition, it has been proven that in leukemia cells, intracellular HMGB1-triggered ferroptosis also engaged two other proteins, transferrin receptor (TFRC) and advanced glycosylation end-product specific receptor (AGER) [3,31]. However, the link between AGER, TFRC, and HMGB1 in the induction of ferroptosis requires further explanation [3]. The voltage-dependent anion channel (VDAC) also appears to be a positive regulator of ferroptosis as it is a direct target for erastin. Knockdown cells in the *VDAC2/3* gene are less sensitive to erastin-induced ferroptosis, while cells with increased expression of this gene appear to be more susceptible to this process [3,32]. Similarly, knockdown of the *CARS* gene (encoding cysteinyl-tRNA synthetase) inhibited erastin-induced ferroptosis, and CARS overproduction made cells sensitive to ferroptosis. Cystine deprivation was indicated as one of the elements that can link CARS level modulation and the induction of ferroptosis [3,35]. The exact mechanisms of the described phenomenon, however, remain to be elucidated for both these genes. The role of pannexin 1 in the induction of ferroptosis in the renal ischemia/reperfusion injury model has also been studied. Reduced tubular ferroptotic cell death was observed in mice with deletion of the *PANX1* gene (encoding pannexin 1) compared to wild-type mice after renal ischemia/reperfusion injury. In addition, silencing of *PANX1* expression in cultured human kidney 2 (HK-2) cells was observed to reduce the severity of ferroptosis (as measured by reduced lipid peroxidation and lower iron levels) after incubation in the presence of erastin [30]. It is also worth emphasizing another role of the p53 protein in the regulation of ferroptosis, apart from that already mentioned above. It was indicated that besides interaction with the SLC7A11 and p21 proteins, p53 can interact with another 11 proteins that may affect ferroptosis. Among them, there are products of the following genes: *GLS2*, *SAT1*, *CDKN1A*, and *DPP4* [36]. By regulating the expression of *GLS2*, p53 increases levels of glutaminase 2 (GLS2), the key enzyme involved in the conversion of glutamine to glutamate. Increased GLS2 levels facilitate glutamine metabolism and lower ROS levels, inhibiting ferroptosis. To confirm this hypothesis, experiments were carried out under conditions of silencing the expression of both *p53* and *GLS2,* and an increase in the level of ROS was observed. These studies were performed on six tumor cell lines [36,37]. Another protein upregulated by p53 is diamine acetyltransferase 1 (SAT1) which contributes to lipid peroxidation and ROS-induced ferroptosis. It was demonstrated that this process is dependent on arachidonate 15-lipoxygenase (ALOX15) because inhibition of its activity resulted in a decrease, and overproduction caused an increase, in the sensitivity of oncogenic Ras-expressing cancer cells to erastin- and RSL3-induced ferroptosis [38].

A mechanism for action of p53 in modulating ferroptosis, different from that by regulating transcription, has been observed in human colorectal cancer cells treated with erastin. This mechanism is related to the DPP4 protease (dipeptidyl-peptidase-4), which, under conditions of a lack or low level of p53, forms a complex with the NOX protein (DPP4–NOX), accelerating lipid peroxidation and entrance of cells into the ferroptotic pathway. High levels of p53 protein lead to more efficient DPP4–p53 complex formation, which antagonizes ferroptosis under these conditions. This is a completely different role of p53 in modulating the efficiency of ferroptosis than those described so far and based on the activity of p53 as a transcription factor [39].

## 3. Implication of Autophagy Process in Ferroptosis (Autophagy-Dependent Ferroptosis)

**Figure 5:**
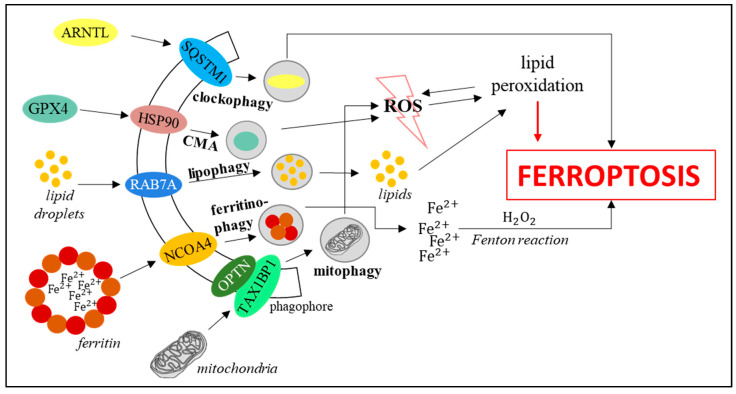
Activation of autophagy-dependent ferroptosis [3,40,41,42,43,44].

In recent years, the dependence of ferroptosis on autophagy was discovered, which led to distinguishing autophagy-dependent ferroptosis. The dependence of ferroptosis on autophagy is based on selective kinds of the latter process, i.e., specific lysosomal degradation of particular proteins of organelles which leads to the modulation of ferroptosis efficiency. The selective autophagy processes which influence ferroptosis include (i) ferritinophagy, (ii) lipophagy, (iii) mitophagy, (iv) clockophagy, and (v) chaperon-mediated autophagy. Molecular mechanisms linking both processes are still under investigation [3]. Ferritinophagy, mediated by cargo receptor NCOA4, localized in the forming autophagosome membrane, contributes to ferroptosis induction due to ferritin degradation and iron liberation. This results in enhancement of the Fenton reaction, followed by lipid peroxidation and cell death. On the contrary, decreased levels of NCOA4 inhibited ferritin degradation, preventing ferroptosis [40]. Another example of autophagy–ferroptosis’ relationship is lipophagy induction, i.e., the degradation of lipid droplets (mediated by cargo receptor RAB7A) to form free fatty acids which can be oxidized. Enhanced formation of lipid droplets due to the upregulation of tumor protein D52 (TPD52) prevented ferroptosis induction due to the limitation of lipid peroxidation [41]. On the other hand, an elevated level of RAB7A caused lipophagy activation, and thus, stimulation of lipid peroxidation-mediated ferroptosis [41]. Most cellular ROS derive from mitochondria. Their production is enhanced when mitochondria are damaged or dysfunctional. The role of mitophagy, i.e., selective degradation of mitochondria, is to remove dysfunctional organelles and to decrease ROS levels, preventing lipid peroxidation and reducing ferroptosis efficiency. Until now, over 10 cargo receptors taking part in mitophagy have been identified, including SQSTM1, OPTN, CALCOCO2, TAX1BP1, and others [3]. Recently discovered selective degradation of the ARNTL protein (aryl hydrocarbon receptor nuclear translocator-like), called clockophagy, can also modulate ferroptosis. Clockophagy-mediated decrease in the ARNTL level results in negative regulation of the transcription factor HIF1, responsible for the stimulation of expression of genes whose products are involved in the transport and binding of fatty acids and lipids (mainly FABP3 and FABP7). Lower levels of these proteins (due to HIF1 deficiency) prevent the binding of fatty acids and lipids and their transportation from the cellular membrane to mitochondria, facilitating their peroxidation and thus, stimulating ferroptosis [42]. In chaperone-mediated autophagy, Hsp70 is employed to direct degradation of proteins with the KFERQ amino acid motif. GPX4 is one of such proteins that plays the role of an antioxidant and protects cells against ferroptosis. Thus, chaperone-mediated autophagy of GPX4 results in ferroptosis stimulation [43,44]. All autophagy-dependent ferroptosis’ induction pathways are presented in Figure 5.

The pathways linking autophagy and ferroptosis appear to be very complex and are only at the early stages of studies. Reports that have already been published provided compelling evidence that autophagic pathways may shift to ferroptotic pathways, and this process can be cargo receptors-dependent. Evidence for this is provided by the fact that lowering the level of cargo receptors led to a reduction in the efficiency of autophagy-dependent ferroptosis [3,40,41,42,43,44]. Almost all studies conducted so far indicated autophagy as the initial process that turns into ferroptosis. For this reason, manipulating the intensity of autophagy is already indicated as the key to anti-cancer therapies, not the other way around [45]. However, there are also recently published reports indicating a reverse relationship. The use of ferroptosis activators (artesunate and erastin) led to the induction of autophagy (measured by the number of accumulated autophagosomes and an increase in the level of the LC3-II protein in MEF cells). Moreover, this effect was not observed in cells with a deletion of the Atg5 protein-coding gene, which indicated that this process is mediated by proteins associated with autophagy [46]. It has also been reported that treatment with erastin led to cancer cell death caused by the upregulation of genes encoding autophagous proteins (Beclin-1, Atg5, Atg12, LC3-II, p62). Under conditions of both silencing their expression and lowering iron levels, erastin-induced cell death was not observed. This would suggest an involvement of both autophagy and ferroptosis in cancer cell death [47]. Thus, one can suggest either a two-way action of ferroptosis activators (which would induce autophagy at the same time) or a feedback linkage between ferroptosis and autophagy (initiated by ferroptosis). However, the exact mechanism of the transition from ferroptosis to autophagy has not yet been proposed.

## 4. Links in the Ferroptosis Induction Network

**Figure 6:**
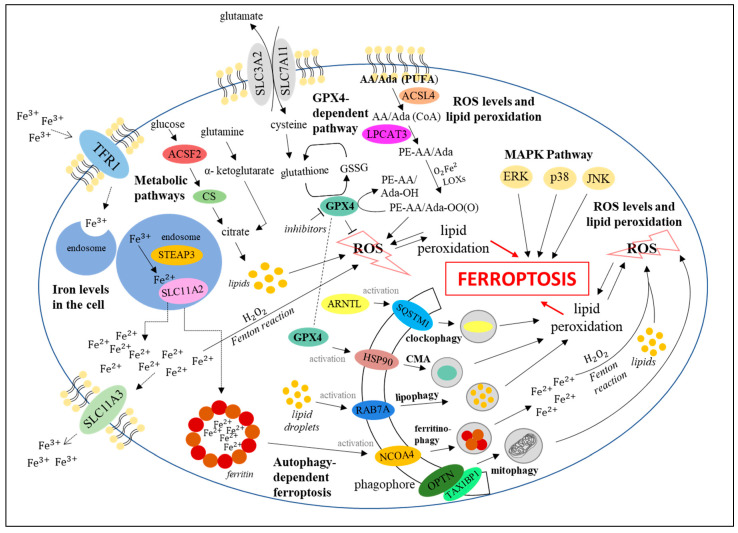
Links in the ferroptosis induction network [1,3,4,16,17,19,20,21,29,40,41,42,43,44].

As indicated in the two preceding sections, there are multiple processes which activate ferroptosis. Particular pathways may lead to ferroptosis independently; however, there are also links between them, forming a specific metabolic network [48]. An example of an interconnection between these pathways is the export of iron (which is stored in a ferritin-bound form) from the endosome to the cytoplasm, mediated by SLC11A2. Increased ferritinophagy, a kind of autophagy leading to the degradation of ferritin, may contribute to the release of large amounts of Fe^2+^ ions, and thus, to stimulation of the Fenton reaction leading directly to lipid peroxidation [3,4]. Another example is the GPX4 protein which plays a crucial role in maintaining the antioxidant status in the cell, while it is also involved in autophagy-dependent ferroptosis, through activation of HSP90 which results in positive regulation of chaperone-mediated autophagy (CMA) and subsequent lipid peroxidation. It is also worth paying attention to aerobic metabolism [1,19,43,44]. Under conditions of its increased efficiency, release of large amounts of ROS from mitochondria might occur, which contributes to the increased intensity of ferroptosis, also in interaction with components of other pathways [3]. Regardless of the starting point of the signal leading to ferroptosis, all these pathways ultimately lead to the ROS-dependent lipid peroxidation that induces ferroptosis. Therefore, the network of processes demonstrated in Figure 6 indicates the complexity of mechanisms of ferroptosis stimulation, and points to complicated regulations of cellular responses to various factors and agents which may influence the activation or inhibition of ferroptosis.

## 5. Ferroptosis Disorders as a Mechanism for Pathogenesis of Lysosomal Storage Diseases

Induction of ferroptosis has been investigated, to date, predominantly in light of anti-cancer therapy. Ferroptosis activators cause cell death in various cancers which is promising in light of the development of anti-cancer therapies [1,2]. However, enhanced ferroptosis has been reported in many other diseases, including Alzheimer’s and Parkinson’s diseases, acute kidney failure, liver and heart injury, ischemic reperfusion injury, and atherosclerosis. Treatment with ferroptosis inhibitors caused increased survival of cellular and/or animal models of these diseases [1,2].

A role for ferroptosis, in both physiological and pathological conditions, is still poorly understood and is the subject of intensive studies. Overactivation of this process appears to worsen the course of all diseases tested to date. On the other hand, it was demonstrated that ferroptosis is required for cell proliferation, which might suggest a physiological role for this process [49,50]. The recently discovered dependence of ferroptosis on the autophagy process, in which the lysosomal system is involved, put our attention on LSD. In fact, changes in ferroptosis efficiency arising from modulation of lysosomal activities have been suggested [3]. As mentioned in the introduction, various therapeutic strategies for LSD have been tested; however, apart from the non-neuronopathic type of Gaucher disease, no abolition of symptoms could be achieved in severe and neuronopathic forms of these diseases, and only partial improvement could be achieved in milder forms [51,52,53].

Reports on disturbances of typical ferroptosis markers and other factors influencing them directly appeared quite long ago in the context of LSD, but so far, they have not been associated with possible ferroptosis disorders. It was only due to the discovery of the pathways linking autophagy and ferroptosis that they, once again, became the factors of pathogenesis of these diseases considered in a new aspect.

### 5.1. Modulation of Iron Levels in Lysosomal Storage Diseases

The first information on iron modulation came from data obtained from patients suffering from neuronal ceroid lipofuscinoses, one of the progressive neurodegenerative LSDs, characterized by excessive accumulation of lipofuscins. These data indicated that the concentration of free iron in cerebrospinal fluids from patients is elevated and increases with disease progression [54,55].

A study on another LSD, Niemann–Pick disease, in which sphingomyelin accumulates in cells, carried out shortly thereafter, showed the surprising result of a complete absence of ferritin, an iron-storing protein, in the visceral organs of four patients [56]. A year later, these results were independently confirmed in more patients, as well as in more biopsy-derived material [57,58]. However, studies on the mouse acid sphingomyelinase-deficient model showed increased levels of the ferritin light chain transcript as well as increased levels of iron in the lungs and brains of animals. These abnormalities were corrected after application of enzyme replacement therapy [59]. If the data on increased mRNA levels for ferritin were confirmed in patients with Niemann–Pick disease, it would mean that the absence of ferritin, as the end product of gene expression, results from translation changes or accelerated protein degradation. Different results were obtained in experiments with the *Npc1*^−/−^ mouse model. They showed a lower iron content in the liver of mice and decreased expression of ferritin light chain and ferroportin, as well as increased expression of the gene encoding the transferrin receptor in the liver of *Npc1*^−/−^ mice of different ages [60]. The differences in the analyses performed may result from using different organs as the research material. This hypothesis might be supported by subsequent studies carried out at a later date on the same model, indicating a significant iron load in the brain of mice, which could potentially contribute to neurodegeneration. The authors also hypothesized the effectiveness of deferiprone, a known brain iron chelator, in improving the pathology of Niemann–Pick disease. However, treatment with deferiprone did not bring the expected results, without affecting the course of the disease or the life span of the mice [61].

Increased iron levels have also been found in liver biopsy samples taken from patients with Gaucher disease, one of the most common LSD [62]. These results were confirmed in studies with a large number of patients, showing not only iron but also ferritin overload [63,64,65]. Magnetic resonance imaging allowed precise localization of excess iron in a group of 40 patients in the liver, spine and femoral bone marrow, and spleen, which correlated with increased serum ferritin levels [66]. This problem is more and more often described in the context of the risk of liver fibrosis [66,67,68] or cancer [66,68]. Subsequent studies indicated not only hyperferritinemia and iron accumulation in patients with Gaucher disease, but also the influence of enzyme replacement therapy on these pathological factors. Treatment with the enzyme resulted in a decrease in hyperferritinemia, increased levels of transferrin, and, most importantly, increased levels of hepcidin, a peptide that regulates serum iron levels. The authors confirmed these results by creating a Gaucher disease cell model and by observing the gradual restoration of ferroportin and hepcidin levels from the time of induction of the disease with a glucocerebrosidase inhibitor in macrophages [69]. The effectiveness of ERT in restoring the correct level of ferritin is also evidenced by recently published case reports [70].

Detailed studies on iron metabolism in Gaucher disease were performed, showing not only the deregulation of iron recycling and modulation of ferritin levels, but also the related release of cytokine and the inflammatory response of the organism [71]. The authors indicated that lowering the iron levels by 4-month therapy with its chelators resulted in a decrease in the concentration of iron in the liver and serum ferritin, and also positively influenced the patients’ response to the available treatment, significantly improving their quality of life [71]. An association of inflammatory reaction with iron metabolism was also found by researchers, which indicated hyperferritinemia and elevated levels of TNFα and some interleukins in over 80% of patients with Gaucher disease in the Swedish population [72].

Studies on iron levels in the brain of patients were also carried out in the case of mucopolysaccharidosis type III (Sanfilippo disease), one of the lysosomal storage diseases in which there is storage of heparan sulfate oligosaccharides. Magnetic resonance imaging showed significant iron accumulation in the deep brain nuclei in two siblings with cognitive impairment [73]. Moreover, an attempt was made to discover the mechanism linking inflammation with iron levels in MPS III. In this disease, extensive neuroinflammation is also observed due to the activation of microglia and astrocytes to produce inflammatory cytokines, in which heparan sulfate is involved. In mice with Sanfilippo disease, iron accumulation and elevated levels of hepcidin (a hormone playing a key role in the regulation of iron homeostasis) have been observed, mainly in the cerebral cortex. The authors also indicated a reduced concentration of ferroportin (a protein that exports iron from cells), which contributed to an increase in iron levels. The in vitro studies showed unequivocally that the accumulation of heparan sulfate directly contributes to the above-described phenomena (increased hepcidin levels and decreased ferroportin levels), and that astrocytes and microglia (as opposed to neurons) are the most vulnerable. Cell signaling studies proved the involvement of the TLR4 and STAT3 signaling pathways in the increase in hepcidin concentration, and thus, iron accumulation in cells [74].

Mucolipidosis type IV is one of the LSD caused by mutations in the *TRPML1* gene, encoding one of the intracellular late endosomal and lysosomal ion channel proteins. Until recently, the divalent metal transport protein SLC11A2 was thought to be the only known endosomal Fe^2+^ transporter. Recent findings indicated that the TRPML1 channel can also transport iron. Mutations in the gene coding for this protein led to an increase in iron levels in late endosomes and lysosomes and a decrease in cytosolic iron, which correlated very well with the severity of symptoms in patients with this disease [75,76].

In mouse models of gangliosidosis type I and type II, diseases caused by the accumulation of lipids known as gangliosides, a significant decrease in iron level was observed in the brain [77]. The authors admitted that this result is in stark contrast to the typically elevated levels of iron found in other LSDs. In order to explain the possible mechanism of this phenomenon, they indicated a decrease in the level of transferrin and an increase in the level of hepcidin. Despite disturbances in the levels of proteins influencing iron metabolism in the brain, administering this element to mice prolonged their life by up to 40% and allowed for a smoother disease transition [77].

The reverse discovery was made with fucosidosis. A case report describing a girl with this condition indicated a significant increase in the level of iron in the brain imaged with the use of magnetic resonance imaging, pointing to fucosidosis as another LSD in which changes in iron metabolism, and thus in ferroptosis, may play a significant role in the pathogenesis of the disease [78].

### 5.2. Lipid Peroxidation in Lysosomal Storage Diseases

The first reports indicating that iron overload may lead to increased lipid peroxidation and, more importantly, that lysosomes may be involved in this phenomenon, appeared already in the 1960s [79]. Those studies already indicated the need to pay attention to the role of disturbances in iron metabolism and lipid peroxidation in the pathogenesis of LSD.

One of the lysosomal diseases in which this problem was highlighted was, as in the case of iron overload, neuronal ceroid lipofuscinosis. Biochemical analyses of patient samples indicated decreases in levels of phosphatidyl ethanolamine polyunsaturated fatty acids (PUFA) and antioxidants [80]. In a canine model of neuronal ceroid lipofuscinoses, an increase in the levels of lipid peroxidation products was also observed [80,81]. An attempt was made to find the cause of ceramide deposition in the tissues of patients, in light of these results. However, no direct evidence of such disease pathogenesis mechanism has been presented.

Studies focusing on lipid peroxidation disorders resulting from changes in the levels of ferritin (which is an antioxidant) also concerned Niemann–Pick disease. The so far described lack of ferritin in patients led to the supposition that an excess of unbound iron could lead to excessive lipid oxidation in ROS-dependent reactions [33]. Studies on this aspect of pathogenesis of Niemann–Pick disease were carried out on fibroblasts collected from patients. Both the concentration of reactive oxygen species and the lipid peroxidation status were increased in these cells compared to cells taken from healthy controls. Moreover, it was noted that the patient-derived fibroblasts were more likely to die from oxidative stress-induced apoptosis. The authors pointed to the participation of NF-κB-dependent signaling pathways in this phenomenon. Moreover, it was shown that silencing the expression of the *NPC1* gene in two cell models led to an increase in ROS concentration [82].

Attempts were also made to find pathways linking excessive lipid peroxidation with the storage of various materials in lysosomes in cystinosis, one of the diseases manifested by abnormal accumulation of the amino acid cystin. Studies conducted on a pharmacologically induced rat model have shown lipoperoxidation and carbonylation of proteins, as well as an increase in the activity of oxidative enzymes such as superoxide dismutase, glutathione peroxidase (GPx), and catalase in the kidneys of animals. In addition, administration of cysteamine, used in the treatment of cystinosis, to rats partially alleviated the proven lesions [83]. Interesting research on cystinosis was also carried out on a model of proximal tubular epithelial cells from patients with this disease, in which the authors confirmed disturbances in oxidative status. They also studied the influence of cysteamine on glutathione level and ATP metabolism, thanks to which they indicated increases in glutathione level and restored glutathione redox status in cystinosis cells [84].

Quite extensive research has been carried out on the aspect of lipid peroxidation in models of mucopolysaccharidoses, a group of LSD in which glycosaminoglycans are the stored material. The first such study, indicating a significant role of oxidative stress in the pathology of the disease, was carried out on patients with MPS I. The level of some markers of oxidative stress was assessed in samples from patients at different stages of enzyme replacement therapy (ERT). The results indicated excessive lipid peroxidation, which gradually normalized with the duration of therapy [85]. Similar studies on the same type of MPS were performed with a mouse model [86]. The authors showed an increase in the activity of the enzymes superoxide dismutase and catalase in some organs, such as the cerebellum, lungs, and spleen. Moreover, in these mice, an increased number of carbonyl groups was noted [86]. Studies performed on a small group of patients with MPS type II showed reduced levels of superoxide dismutases and glutamate transporters. However, no changes in the lipid peroxidation process were found [87]. Extended studies on MPS II patients were performed, where oxidative stress parameters were compared in the blood of patients before and after ERT initiation [88,89]. The authors described decreased levels of antioxidant enzymes and increased levels of nitric oxide in plasma prior to enzyme administration. In addition, increased levels of nitrates and nitrites in the urine of patients have been observed. A similar analysis after a few months of therapy showed a significant reduction in the level of malondialdehyde and an increase in the level of sulfhydryl groups. There were no changes in the activity of catalase, superoxide dismutase, glutathione peroxidase, and glutathione reductase. These results indicated a significant exposure of patients with MPS II to the oxidative damage of lipids and proteins and showed a reduction in the amount of antioxidant enzymes [88,89]. Research on oxidative stress was also performed on the MPS IIIB mouse model [90]. Mice at various stages of the disease demonstrated extensive oxidative stress, affecting the CNS from the very first months of life. Additionally, it has been proved that the processes of lipid peroxidation, and protein and DNA oxidation concerned mainly the cerebellum. The authors also showed changes in the expression of genes encoding enzymes involved in oxidation, such as *Sod1*, *Ret*, *Bmp4*, *Tgfb*, *Gzmb*, and *Prf1*, were already at the level of their transcripts. These data indicate, on the one hand, the newly discovered mechanisms leading to the induction of oxidative stress in MPS, and on the other hand, that they appear at a very early stage of the disease, even before symptoms develop [90].

Markers of oxidative stress were also noted in the case of Fabry disease, manifested by an accumulation of glycosphingolipids in cells. Studies carried out on samples taken from patients showed increased lipid peroxidation, and increased levels of nitric oxide and glutathione, as well as decreased levels of glutathione peroxidase and heme oxygenase [91,92]. In patients undergoing enzyme replacement therapy, lipid peroxidation status and nitric oxide levels remained elevated. These results indicated that despite the therapy, oxidative status disturbances were still observed in patients, which may suggest that lipid peroxidation disorders are independent pathogenic factors of this disease [91]. The authors proposed a strategy of inhibiting oxidative stress by pharmacological or nutritional measures as an adjunctive therapy to enzyme replacement therapy in Fabry disease [92].

Other examples of LSD, in which increased lipid peroxidation, ROS accumulation, increased transcription of genes responding to cytotoxic oxidative stress, and decreased levels of antioxidant enzymes were observed, include mucolipidosis type IV (studies on TRPML1-knockdown cells) [93] and Gaucher disease (studies performed on red blood cells collected from patients) [94].

The latest reports also mentioned disturbances in oxidative status in a murine model of Krabbe disease, characterized by the accumulation of psychosin. In *twi*^−/−^ mice, increased lipid peroxidation and decreased levels of antioxidant enzymes were observed. Supplementation with vitamin D3, as a known antioxidant, resulted in increased expression of genes encoding antioxidant enzymes, decreased lipid peroxidation, decreased inflammation, and delayed psychosin accumulation, which ultimately increased axon integrity in the brain of animals [95].

### 5.3. Modulation of Activity of the GPX4-GSH-Xc^−^ System in Lysosomal Storage Diseases

Parkinson’s disease is the first neurological disease in which modulation of GPX4 levels has been described. In the case of this disease, both increased oxidative stress and iron accumulation were observed, which may lead to the intensification of the phenomenon of ferroptosis. An increase in expression efficiency of the gene encoding GPX4 has been demonstrated in both in vitro and in vivo models [96]. Changes in glutathione and ROS metabolic pathways have also been studied in myocardial infarction. Studies conducted on a mouse model of this ailment showed a decrease in glutathione metabolism and an increase in ROS levels. One of genes with the most depressed expression was *GPX4*. The authors confirmed the observed changes by creating a model of H9c2 cardiomyoblasts with silenced expression or depletion of *GPX4* in which they observed accumulation of lipid peroxide leading to ferroptotic cell death [97].

In the context of lysosomal storage diseases, there are few reports of modulation of the GPX4-GSH-Xc^−^ system. It is worth paying attention to more and more reports pointing to the connection of the activity and levels of this system to the functions of lysosomes [19,98,99]. However, there are results of experiments indicating the reduction in the level of glutathione peroxidase in the *Cln3* knock-in (*Cln3* (*Deltaex7/8*)) mouse model of neuronal ceroid lipofuscinosis [100]. Decreased activity of this enzyme was also noticed in urine and blood samples of patients suffering from Fabry disease, even though those patients were treated with enzyme replacement therapy [101]. Studies on activities or levels of antioxidant enzymes in LSD have typically involved assessing redox potential/oxidative status as an aspect influencing cell dysfunction. More reports indicated a modulation of GSH levels. A decrease in GSH level was observed mainly in cystinosis, as the decrease in the level of cysteine, as one of the substrates for GSH synthesis, is the main cause of these diseases [102,103]. It is worth noting that the decreased level of intracellular cysteine may be the result of dysfunctions of lysosomes which ineffectively degrade macromolecules (especially in LSD), and which in turn may lead to a reduction in GSH levels [19]. Reduced GSH levels have been reported not only in cysteinosis but also in urine and blood samples from patients with Fabry disease and mucopolysaccharidosis type IVA. It is worth noting that in these studies, the investigated patients were treated with enzyme replacement therapy, and yet the levels of GSH remained lower relative to the group of healthy persons [101,104]. These results were confirmed in experiments with the Fabry disease mouse model [105]. This may indicate that the pathogenesis of LSDs is not limited to the primary cause—accumulation of the storage material. Such a hypothesis can be corroborated by recently published results demonstrating dysregulation of expression of hundreds of genes, coding for proteins involved in various cellular processes, in fibroblasts derived from patients suffering from mucopolysaccharidoses [51,106,107,108,109,110,111]. 

A summary of the modulations of ferroptosis features in LSD is presented in Table 2, with ferroptosis markers defined as features that are necessary and sufficient for the ferroptotic process to take place.

### 5.4. The Effectiveness of Ferroptosis Modulators in the Course of Lysosomal Storage Diseases: Already Published Reports and Perspectives

An obvious question appeared if there is a potential efficacy of ferroptosis modulators in abolishing or at least alleviating the symptoms of genetic diseases, including LSD. Reports on the potency of ferroptosis inhibitors in human diseases are contradictory. Research on the effectiveness of ferrostatins in Huntington’s disease, periventricular leukomalacia, and kidney proximal tubules cellular models showed an increase in their viability [112]. However, studies on a number of ferroptosis inhibitors in the Parkinson’s disease nerve cell models indicated that only one of them (liproxstatin) was effective in reversing cell death [113]. 

Similarly, divergent results have been obtained for LSD. Studies on the effectiveness of deferiprone (iron chelator, crossing the blood–brain barrier) showed no changes in the trajectory of the disease and life expectancy in mice with Niemann–Pick disease [61]. On the other hand, iron chelators, deferoxamine or deferasirox, have also been used in patients with Gaucher disease. Short-term drug intake led to a significant reduction in serum ferritin and hepcidin, and iron deposition in the liver. Patients’ clinical and analytical data also improved after long-term follow-up [71].

However, one should be aware that mechanisms of ferroptosis induction are very complicated, as underlined also in this article. These mechanisms may be different in different tissues and organs, and in the case of changes in cell physiology (such as in human diseases), various pathways leading to ferroptosis might be disturbed. This may result in the ineffectiveness of individual ferroptosis inhibitors. Certainly, this aspect requires further research.

## 6. Autophagy Disorders in Lysosomal Storage Diseases

Disorders of the autophagy process have already been described quite extensively in the context of the pathogenesis of LSD [114,115,116]. The discovery of autophagy-dependent ferroptosis, however, sheds new light on this process. Thus, autophagy should be recognized not only as a cellular process that is altered in performance with LSD, but also as a process that can lead to cell death.

In most of the LSDs described so far, various phenotypes of autophagy have been observed. They can take place at various stages, from autophagosome formation and maturation, through the accumulation of abnormal autophagosomes, to autophagic flux blockage. Such disorders have been observed so far in neuronal ceroid lipofuscinoses, glycogenosis, Niemann–Pick disease, Gaucher disease, mucolipidosis type IV [114], Fabry disease [115], and mucopolysaccharidoses [51].

## 7. Possible Role of Autophagy in Ferroptosis Modulation in Lysosomal Storage Diseases

Autophagic flux disorders due to lysosomal dysfunction in LSD are inevitable. One of their consequences may be the accumulation of autophagosomes, the appearance of which may induce cellular stress and initiate cellular processes to deal with them. Unfortunately, the accumulation of autophagosomes can trigger further pathological cellular phenomena. The recent discovery of autophagy-dependent ferroptosis points to the autophagosome as the source of cargo receptors, initiating the response leading to ferroptotic cell death. Due to the relatively recent discovery of this way of ferroptosis induction, reports on the modulation of levels of cargo receptors in LSD are so far infrequent and concern a few diseases, but their number is growing rapidly. Some of the popular proteins found to be cargo receptors in autophagy-dependent ferroptosis have already been studied in LSD because of their other important cellular functions, such as involvement in lysosomal and proteasomal degradation (SQSTM1) or endocytosis (Rab7A). The rest remains an open question.

The greatest number of LSDs in which the levels of the proteins mentioned above were measured were indicated for SQSTM1. Studies on mucolipidosis type IV in a model of *Mcoln*^−/−^ mouse have indicated inclusions formed by SQSTM1 in animal brains [116]. For the same disease, similar results were shown in the *Mcoln1*^−/−^ neuronal culture model [117]. Moreover, in mucolipidosis type II and III, an increase in the levels of the described protein [118] was indicated in studies on the model of fibroblasts taken from patients. The accumulation of SQSTM1-positive aggregates has also been demonstrated in glycogen storage disease type II [119], nephropathic cystinosis [120], and Gaucher disease [121]. For another cargo receptor, Rab7A, one more LSD, Niemann–Pick disease, was tested. The studies showed an increase in the level of this protein in the liver of the *Npc1*^−/−^ mice [122]. In all these studies, the authors set the primary goal of research on the effectiveness of autophagy or proteasomal degradation or endocytosis. Of course, the accumulation of SQSTM1 in cells could be the result of the response to the appearance of misfolded proteins, and the accumulation of Rab7A could mean a serious disturbance in endocytic transport. However, at that time, no one imagined that these proteins could also play a key role in cell death.

## 8. Concluding Remarks

Disturbances in iron homeostasis, lipid peroxidation, as well as the efficiency of the autophagy process and cell death, have been observed for years in LSDs. However, the recent discovery of autophagy-dependent ferroptosis not only drew attention to the problem of ferroptosis as one of the causes of neurodegeneration in LSD (due to the known dysfunction of lysosomes in these diseases) but also led to the assumption that there are pathways that connect the described phenomena, observed in these diseases, and may partially explain the pathophysiology of neurodegeneration. In this paper, we hypothesize that the accumulation of autophagosomal vesicles resulting from a decrease in autophagy efficiency in LSDs may initiate the process of ferroptosis in an autophagy-dependent manner. The so far proven elevated levels of some cargo receptor proteins in the initiation of ferroptosis (SQSTM1, Rab7, and certainly also others) may initiate a number of reactions which result in an increase in iron concentration or oxidative stress, which are the basic markers of ferroptosis, occurring in most of these diseases. Therefore, this work points to autophagy-dependent ferroptosis as one of the additional LSD pathomechanisms that can lead to cell death.

It is worth noting, however, that the presented modulations of the level or activity of features typical for ferroptosis were studied in LSD in a completely different context than the study of its role in the pathogenesis of this group of diseases. The idea that ferroptosis disorders are possible additional aspects of LSD pathology, presented in this paper and based on the recently discovered autophagy–ferroptosis relationships, and the modulation of its markers determined so far, is therefore a novel hypothesis. In fact, there are some reports suggesting that possible ferroptotic disorders may contribute to the pathogenesis of this group of diseases, though they were not devoted to understanding LSD–ferroptosis connections. Taken together, those reports did not provide conclusive evidence for a strong relationship between LSD and ferroptosis; however, they led us to propose the above presented hypothesis of which verification will be necessary in the near future.

## Figures and Tables

**Table 1:** Comparison of major features of apoptosis, autophagy, and ferroptosis (based on [1]).

Features		Apoptosis	Autophagy	Ferroptosis
morphology	cell membrane	plasma membrane blebs forming	no changes	cell rounding up (lack of plasma membrane blebs)
	cytoplasm	pseudopods retraction and cellular volume reduction	accumulation of autophagosomes and autophagolysosomes	reduction in size of mitochondria, condensed densities of mitochondrial membrane, rupture of the mitochondrial membrane, reduction in mitochondrial cristae
	nucleus	nuclear volume reduction, fragmentation of nucleus and chromatin condensation	lack of chromatin condensation	lack of nuclear volume reduction and chromatin condensation
biochemistry		caspases activation, DNA fragmentation, exposure of phosphatidylserine, mitochondrial membrane potential (ΔΨm) dissipation	conversion of LC3-I to LC3-II form, increased levels of the LAMP2 protein, increased levels of Atg proteins and the Beclin-1 protein	reactive oxygen species accumulation, lipid peroxidation, iron accumulation, activation of MAP kinases, inhibition of cystine-glutamate antiporter, increased NADPH oxidation, glutathione depletion, arachidonic acid mediators, mitochondrial membrane potential (ΔΨm) dissipation

**Table 2:** Modulations of ferroptosis features in lysosomal storage diseases.

Ferroptosis Marker		Disease	Model	Material/Organ	Reference(s)
increased iron concentration		NCL	Patients	cerebrospinal fluid	[54,55]
		Niemann–Pick Disease	ASMKO mice	lung and brain	[59]
			BALB/cJ Npc1nih (*Npc1*^−/−^) mice	brain	[61]
		Gaucher disease	Cells	patient-derived skin fibroblasts	[62]
			Patients	liver or spleen biopsy serum MRI imaging of liver, bone, marrow, spleen	[62,63,67] [64,65,69] [66,71]
		MPS type III	Patients	MRI imaging of brain	[73]
			C57Bl/6 *Naglu*^−/−^ mice	brain	[74]
		ML type IV	Cells	patient-derived skin fibroblasts	[75]
		Fucosidosis	Patients	MRI imaging of brain	[78]
increased lipid peroxidation		NCL	Patients	serum, brain	[80]
			English setters American bulldogs	brain brain, eyes	[80] [81]
		Niemann–Pick disease	Cells	patient-derived skin fibroblasts	[82]
		Cystinosis	rats loaded with cystine dimethyl ester	kidney	[83]
			Cells	proximal tubule epithelial cells taken from patients	[84]
		MPS type I	Patients	serum	[85]
			C57BL/6 *Idua*^−/−^ mice	brain, heart, lung, diaphragm, liver, kidney, spleen	[86]
		MPS type II	Patients	plasma urine	[88,89] [89]
		MPS type IIIB	C57Bl/6 *Naglu*^−/−^ mice	brain	[90]
		Fabry disease	Patients	blood, urine plasma	[91] [92]
		ML type IV	cells	*TRPML1*^−/−^ cells	[93]
		Gaucher disease	patients	erythrocytes and plasma	[94]
		Krabbe disease	GALC *twi*^−/−^ mice	brain	[95]
inhibition of activity or decreased level of GPX4-GSH-Xc^−^ system components	GPX4	NCL	*Cln3Dex7/8* knock-in mice	brain	[100]
		Fabry disease	patients	urine and blood	[101]
	glutathione (GSH)	infantile cystinosis	cells	patient-derived skin fibroblasts	[102]
		nephropathic cystinosis	cells	patient-derived skin fibroblasts	[103]
		Fabry disease	patients	urine and blood	[101]
			B6/129-B57BL/6 Fabry mice	heart, kidney, liver, plasma	[105]
		MPS type IVA	patients	urine and blood	[104]
	Xc^−^	no data	-	-	-

Abbreviations: NCL—neuronal ceroid lipofuscinoses; ML—mucolipidosis; MPS—mucopolysaccharidosis.

## Data Availability

No new data were created or analyzed in this study. Data sharing is not applicable to this article.
